# Identification of Risk Areas for Intestinal Schistosomiasis, Based on Malacological and Environmental Data and on Reported Human Cases

**DOI:** 10.3389/fmed.2021.642348

**Published:** 2021-08-06

**Authors:** Paulo R. S. Coelho, Fabrício T. O. Ker, Amanda D. Araújo, Ricardo. J. P. S. Guimarães, Deborah A. Negrão-Corrêa, Roberta L. Caldeira, Stefan M. Geiger

**Affiliations:** ^1^Department for Parasitology, Federal University of Minas Gerais, Belo Horizonte, Brazil; ^2^Oswaldo Cruz Foundation (Fiocruz), Research Group on Helminthology and Medical Malacology, René Rachou Institute, Belo Horizonte, Brazil; ^3^Secretaria de Vigilância em Saúde, Instituto Evandro Chagas, Ananindeua, Brazil

**Keywords:** *Schistosoma mansoni*, *Biomphalaria*, malacological survey, risk mapping, ecological models

## Abstract

The aim of the present study was to use an integrated approach for the identification of risk areas for *Schistosoma mansoni* transmission in an area of low endemicity in Minas Gerais, Brazil. For that, areas of distribution of *Biomphalaria glabrata* were identified and were related to environmental variables and communities with reported schistosomiasis cases, in order to determine the risk of infection by spatial analyses with predictive models. The research was carried out in the municipality of Alvorada de Minas, with data obtained between the years 2017 and 2019 inclusive. The Google Earth Engine was used to obtain geo-climatic variables (temperature, precipitation, vegetation index and digital elevation model), R software to determine Pearson's correlation and MaxEnt software to obtain an ecological model. ArcGis Software was used to create maps with data spatialization and risk maps, using buffer models (diameters: 500, 1,000 and 1,500 m) and CoKriging. Throughout the municipality, 46 collection points were evaluated. Of these, 14 presented snails of the genus *Biomphalaria*. Molecular analyses identified the presence of different species of *Biomphalaria*, including *B. glabrata*. None of the snails eliminated *S. mansoni* cercariae. The distribution of *B. glabrata* was more abundant in areas of natural vegetation (forest and cerrado) and, for spatial analysis (Buffer), the main risk areas were identified especially in the main urban area and toward the northern and eastern extensions of the municipality. The distribution of snails correlated with temperature and precipitation, with the latter being the main variable for the ecological model. In addition, the integration of data from malacological surveys, environmental characterization, fecal contamination, and data from communities with confirmed human cases, revealed areas of potential risk for infection in the northern and eastern regions of the municipality. In the present study, information was integrated on epidemiological aspects, transmission and risk areas for schistosomiasis in a small, rural municipality with low endemicity. Such integrated methods have been proposed as important tools for the creation of schistosomiasis transmission risk maps, serve as an example for other communities and can be used for control actions by local health authorities, e.g., indicate priority sectors for sanitation measures.

## Introduction

In 2018, a centenary had passed since the discovery and description of the intermediate hosts of *Schistosoma mansoni* Sambon, 1907 in Brazil by Adolpho Lutz (1). This human parasitic disease, also endemic in other Latin American countries, remains one of the major public health problems in tropical and subtropical regions of the world (2). It is estimated that approximately 240 million individuals are infected with schistosome species and 700–800 million people worldwide are at risk of infection (3). In Brazil, the presence of *S. mansoni*, with its complex life cycle, depends on human beings as main definitive hosts and freshwater, planorbid molluscs of the genus *Biomphalaria* as intermediate hosts. In the field, three snail species were found naturally infected with *S. mansoni*: *Biomphalaria glabrata* (Say, 1,818), *Biomphalaria tenagophila* (Orbigny, 1,835) and *Biomphalaria straminea* (Dunker, 1,848).

In the State of Minas Gerais (MG), schistosomiasis is still endemic, but such substantial progress in the control of the disease has been achieved during the past decades, due to the implementation of the National Schistosomiasis Control Program (NSCP), that the positivity rate fell from 10.1% in 1977 to 3.86% in 2015, during the latest national survey (4). However, even in Brazil, the goal of transmission control or even elimination of schistosomiasis in most of the endemic areas still seems to be remote, considering latest epidemiological data on endemic areas and the vast distribution of intermediate hosts (5). Nevertheless, community and individual parasite loads have dropped during the last decades with more or less regular treatment rounds by NSCP and, nowadays, most individuals from endemic areas present with reduced parasite loads, are hard to detect by common diagnostic methods (6).

In the central region of Brazil, the “Estrada Real” belongs to the old slavery road, which transported diamonds and gold from Northern Minas Gerais to the commercial ports of the states of Rio de Janeiro (RJ) and São Paulo (SP) and, which is very popular today among backpackers and eco-tourists. In addition, it is also known for its risk areas and active transmission of intestinal schistosomiasis, where prevalence rates at the municipal level ranged from 0.06 to 28.2% (7).

One of the smaller municipalities of the “Estrada Real” is Alvorada de Minas, where adequate intermediate hosts were shown to be present (8), but the municipality is considered to be an area of low endemicity by the local health authorities. However, the last NSCP data indicated prevalence rates of between 5.0 to 10.0% (7). At present, the municipality has no active control program and sporadic, individual cases have been treated by the local public health system.

Since the 1970s, NSCP has promoted classic intervention actions that has prioritized large-scale treatment with praziquantel (9), without considering sometimes very different epidemiological settings, intermediate host distribution, or sanitation conditions in the vast endemic areas of Brazil (10). In its resolution WHA65.21, participants of the World Health Assembly in 2012, agreed on the intensification on control measures for several neglected tropical diseases, including schistosomiasis, and on its elimination as a public health problem by 2020 (11). However, as that time approached and passed, it became clear that interventions against schistosomiasis, which were merely based on preventive chemotherapy, most probably, would fall short of achieving the envisaged goal of elimination (5, 12, 13). More precisely, in order to reach such goals, integrated control measures were proposed, including sanitation, health education, early diagnosis and treatment, and monitoring and control of intermediate hosts (5).

In this respect, geo technologies are considered emerging epidemiological tools for monitoring and decision making of integrated control measures against schistosomiasis in Brazil and in any other endemic region of the world (14–25). These tools and their spatial analyses allow the construction of risk maps, which define the epidemiological and spatial dimensions of each disease, information which is crucial to plan and direct control interventions (26, 27).

In the present work, predictive models were created for schistosomiasis risk areas, which were based on intermediate host distribution, presence of coliform bacteria in natural water bodies, and environmental characterization. In addition, and in collaboration with the local health authorities, we combined this data with the notification of schistosomiasis cases in the different communities of the investigated municipality.

## Materials and Methods

### Study Area

The study was conducted in the Municipality of Alvorada de Minas, Minas Gerais State, Brazil (18°43'7” S/43°22'5” W), which is located in the mesoregion “Metropolitan Belo Horizonte” and within the microregion of Conceição do Mato Dentro, about 210 km north of the capital Belo Horizonte. According to the latest survey by the Brazilian Institute for Geography and Statistics, its populations consisted of 3,606 inhabitants and the municipality occupied an area approximately of 375 km^2^ (28). The main economical sources are mining activities, farming, and cattle breeding. The municipality is a mountainous region, partly covered by Atlantic rainforest and with a tropical climate. It is divided by the “Rio do Peixe” river, which is the main water source of the municipality and which has an extension of nearly 165 km on its way to the “Rio Doce” river. Data from 2017 indicated that only 33.8% of houses and other constructed buildings had adequate sanitation (28), which is supposed to be a risk factor for the transmission of schistosomiasis.

### Environmental Epidemiology

#### Malacological Surveys

The malacological surveys were undertaken five times over a 12-month period (starting in September 2018) and covered the dry and rainy seasons. The snails were collected during a collection effort of 20 min at each collection point, as described elsewhere (29). Criteria for collection points were: localities indicated by the health workers; water bodies used by the population for domestic or recreational purposes; proximity to houses (urban or rural), especially in communities with registered human cases; and water bodies with ready road access.

**Figure 1:**
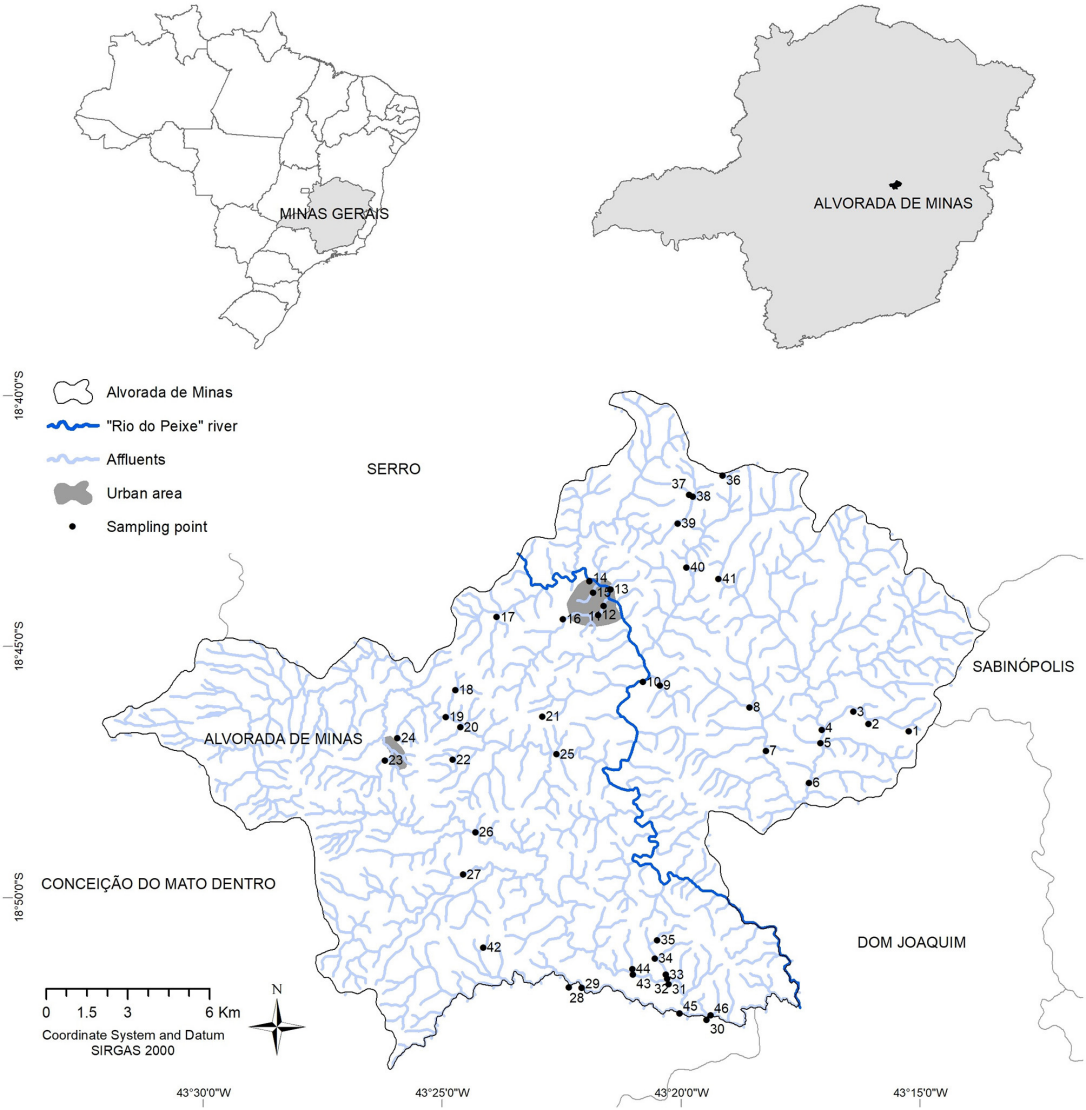
Distribution of water resources and the 46 collection points for the investigation of limnic molluscs, between the years 2018 and 2019, in the Municipality of Alvorada de Minas, Minas Gerais, Brazil.

Captured molluscs were enrolled in humidified gauze, put in identified plastic containers (30), and transferred to the laboratory in Belo Horizonte (Institute for Biological Sciences, Federal University of Minas Gerais). Molluscs were identified by the Schistosomiasis Reference Laboratory of the René Rachou Institute, Fundação Oswaldo Cruz, Belo Horizonte. At each date and point of collection, five percent of sampled *Biomphalaria* were dissected and the taxonomic classification performed by molecular methods PCR-RFLP (Polymerase Chain Reaction-Restriction Fragment Length Polymorphism) with the *Dde*I enzyme. The protocols and profiles chosen were compared to those improved by Vidigal et al., 2000 and Caldeira et al., 2016 (31, 32). For molluscs not identified by molecular methods, the morphological identification procedure of the shells was adopted according to Deslandes, 1951 and Paraense, 1975 (33, 34). Each collection point and water body (river, brook, canal, and lagoon) was georeferenced, using a Garmin GPSMap 62S (Figure 1).

In order to stimulate and verify cercariae release in the laboratory, all specimens of the genus *Biomphalaria* captured were individually exposed to an artificial light source for a duration of 4 h, at a temperature of between 28 and 30°C. Afterwards, the presence of typical, bifurcate cercariae was verified under a stereomicroscope and a 20x magnification (35, 36). Snails without cercarial shedding were re-examined once a week, for a total of 4 weeks. Each analysis contained the confirmation of cercarial shedding during the daytime (light stimulation) and overnight, when molluscs were individually checked for the presence of other types of cercariae in the early morning, in order to certify absence of any trematode infection.

The research was submitted to the Biodiversity Authorization and Information System (Sisbio), under number 68627-1 and authentication code 068627012019029.

#### Fecal Contamination of Water Samples With Coliform Bacteria

The Colilert® test kit (IDEXX Laboratories Inc., Westbrook, Maine, USA) was used for the examination of water samples from different collection sites where molluscs were captured. Additionally, water samples from three urban locations without the presence of molluscs were included, two from the central urban area and one from a district of the municipality, all areas frequently used by the population. The test kit is capable of detecting the presence of coliforms and especially *Escherichia coli* in liquid samples.

For the identification of *E. coli*, it was necessary to use a 365 nm ultraviolet lamp according to (37), for the detection of fluorescence produced in the metabolism of 4-methylumbelliferyl-β-d-glucuronide (MUG) (38, 39).

#### Environmental Characterization

The environmental characterization of the collection sites followed the guidelines for the evaluation of diversity of habitats, as proposed by the Environmental Protection Agency (EPA) (40) with adaptations, as proposed by (41, 42). The protocol evaluated the characteristics of each segment of water body and its environmental impacts, imposed by anthropogenic activities. For a better description of the habitats of the malacofauna, we modified parameters 8 and 10, according to (42) (see Supplementary Material Appendix 1). As proposed in another adaptation for environmental characterization (41), three levels for environmental preservation were defined, where 0–15 points indicated impacted water bodies, 16–25 points altered, and higher than 26 points indicated natural water bodies.

#### Registered Human Cases of Schistosomiasis in Alvorada de Minas

In order to relate the malacological data to the occurrence of human schistosomiasis cases, non-identifiable information on human cases in settlements or communities of each district was obtained from the local Secretary of Health for the years 2017 to 2019 inclusive.

Since 2011, the national program for schistosomiasis control has been discontinued and no sistemic epidemiological surveys have been conducted in this area. At present, suspected cases are being dealt on an individual basis, with fecal and/or serological exams and individual treatment. The municipality has a low endemicity profile for schistosomiasis, with 107 cases between the years 2017 to 2019, with an average of 35 cases per year, <1% in the municipality's population. Human parasitological research in Alvorada de Minas will be carried out at another time in the study, when funding is available.

### Spatial Epidemiology

#### Obtained Data

The ecological study analyzed the spatial patterns of the obtained data. Based on a codified cartography, a database with each of the sampling points, data on present snail species, fecal contamination with *E. coli*, quality of habitat, and presence of human cases in the respective community was mounted. After evaluation and integration of the different variables, the information was converted in nominal values, which contained and defined the arbitrary variable “weight” and served for the identification of risk areas (Table 1).

**Table 1:** Arbitrary score sheet of the variables obtained in the field.

**Variables**	**Assigned score**	**Scoring criteria**
*Biomphalaria glabrata*	2	High susceptibility
Other specimens of *Biomphalaria*	1	Low susceptibility or not susceptible
*E. coli* contamination	1	Presence of *E. coli*
Habitat	1	Attributed to highly impacted environments
Human Infection	1	Presence of patients diagnosed and treated between 2017 and 2019 in the communities.

From the coordinates, the shape of points containing the database was generated. Other related databases were imported: Brazilian municipalities geocoded database, projection and datum considered Geocentric Reference System for the Americas version 2000 (SIRGAS 2000–IBGE, 2018). From these databases, choropleth maps were generated for the analysis of spatial distribution of data in relation to environmental variables.

The remote sensing geo-climatic variables (temperature, vegetation index, precipitation, and digital elevation model) were obtained from the Google Earth Engine (GEE) using the products MODIS/006/MOD11A2, MODIS/006/MOD13Q1, UCSB-CHG / CHIRPS / DAILY, and USGS / SRTMGL1_003, respectively. GEE is a cloud platform used for geospatial analysis, with high-performance computational resources and providing a wide range of data (43). Due to the absence of some data, only the distribution of *B. glabrata* was used for the correlation with the GEE data.

Data on cover and land use in 2018 were extracted from the Brazilian Annual Land Use and Land Cover Mapping Project (Projeto MapBiomas, 2020) (**https://mapbiomas.org/**) version 5.0 (44).

The ecological variables (Climatic, Topography and Vertical Distance to the Nearest Drainage data) were obtained from the National Institute for Space Research (http://www.dpi.inpe.br/Ambdata/), which provides environmental data on biodiversity studies, with a 1 km spatial resolution. The distribution of *B. glabrata* was added to the ecological model.

#### Processing Data

The script (https://code.earthengine.google.com/35ac6e4beae421f8f22d6aaee16df141) was constructed to obtain the image with average values from October 2018 to September 2019, the period corresponding to the malacological collection. From these databases, choropleth maps were generated for the analysis of spatial distribution of data in relation to remote sensing (RS) variables.

Pearson's coefficient was used to verify the existence of a correlation between the presence of *B. glabrata* and environmental and RS variables. For determining the significance of the results, *p* ≤ 0.05 was adopted.

The configurations of MaxEnt used were: cross validate and logistic. The performance of the model was ascertained using the area under the curve (AUC) of the receiver operating characteristic.

To suggest risk areas, geoprocessing techniques and spatial analysis tools were used. The Buffer technique was used to point out potential risk areas from the radial distance from a risk point (45). The hydrographic sub-basins of the municipality were isolated and delimited and buffers of 500, 1,000, and 1,500 m were merged from the sampling points with the presence of *B. glabrata*. The sections of water resources internal to these buffers were cut and highlighted, excluding those sections belonging to other basins, in order to estimate the possible expansion of outbreaks during the rainy periods.

For each point, arbitrary values were determined individually for each study variable, which were later grouped to determine what was classified by us as “Total Arbitrary Weight” (Table 1), which was used to indicate potential risk even without the presence of *B. glabrata* at that point.

The CoKriging Interpolation technique was used to perform the prediction of spatial risk in locations that had not been measured, based on measurements made at specific points (46). The Source Dataset was used in the following order: presence of *B. glabrata* (weight two) with presence of other *Biomphalaria* species (weight one), community with human cases (weight one), altered habitat (weight one), and *E. coli* contamination (weight one). An Ordinary method that presented Semivariogram Model [0.58755 ^*^ Nugget + 0.5771 ^*^ Stable (0.1726, 0.089162, 137.3, 2)], Anisotropy true, Lag Size 0.014; Number of Lags 12.

Geoprocessing and spatial analyses were performed in ArcGis 10.4 (https://www.arcgis.com/), Pearson's correlation was performed using R software (https://www.r-project.org/) and ecological model was performed using MaxEnt software (https://biodiversityinformatics.amnh.org/open_source/maxent/).

## Results

### Malacological Surveys, Fecal Contamination and Its Spatial Distribution

**Figure 2:**
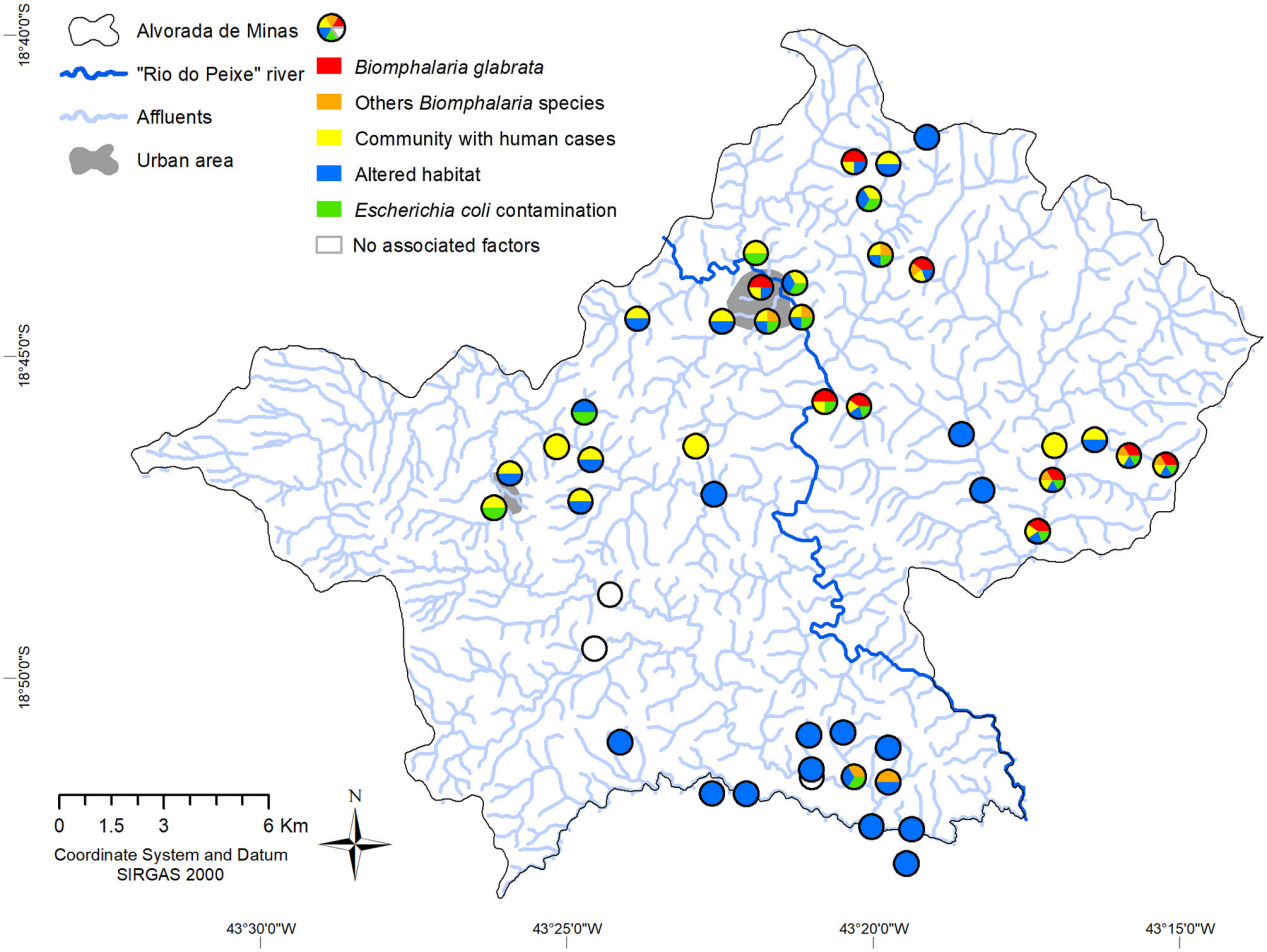
Distribution of environmental and epidemiological data (malacology, altered environments and the presence of *E. coli*), and cases of infected humans in the nearby communities, identified by collection points, in the municipality of Alvorada de Minas, Minas Gerais, Brazil. The white circles represent investigated areas that did not show factors associated with schistosomiasis.

Forty-six collection points were evaluated, of which 14 points contained snails of the genus *Biomphalaria*. In total, 767 molluscs were collected and from morphological and molecular analyses the presence of *B. glabrata, B. tenagophila, B. straminea, B. cousini* and *B. kuhniana* was confirmed. The first three of those are natural intermediate host species of *S. mansoni* and *B. cousini* and are considered potential intermediate hosts, which is based on experimental infections in the laboratory (47). Most of the captured planorbid snails were collected in lentic environments, e.g., in 13 out of 14 collection points, but none of the snails eliminated *S. mansoni* cercariae. *Biomphalaria glabrata* and other species of this genus were found in the urban area and were also distributed in the eastern affluents of the main river (Rio do Peixe), with the exception of two points in the southwestern region, where only other *Biomphalaria* species were captured (Figure 2).

As for the classification of the environments of the different collection points, the applied protocol indicated the presence of 37 (80.4%) highly impacted and 9 (19.5%) slightly impacted environments. Of the environments with the presence of *Biomphalaria*, 13 (92.8%) were classified as highly impacted sections and only one collection point (# 10) was classified as slightly impacted (see Supplementary Material Appendix 2). The environments evaluated as impacted or altered were classified in the spatial distribution as altered habitats, which was the case in 37 of the 46 sampling points, distributed throughout all districts and regions of the municipality (Figure 2).

Microbiological analysis of water quality was limited to 18 environments, with the presence of different limnic molluscs and at points 13, 14, and 23 without registration of any mollusc. Of the 14 environments that presented *Biomphalaria*, microbiological analysis was carried out in 13 environments, and of these, only three collection points (15, 32, and 41) were not contaminated with *E. coli*. Overall, coliform bacteria was found in all water samples 17 (100%) and 15 (88.23%) of the samples were positive for *E. coli* (Figure 2).

Within the municipality, 10 communities were registered with cases of schistosomiasis, which corresponded to 26 collections points for limnic environments (Figure 2). The western district of the municipality (*Itaponhoacanga*) recorded human cases, altered habitat and fecal contamination, but no *Biomphalaria* species were found in this area. In the communities to the south, altered habitats, fecal contamination and *B. tenagophila* were registered, but there was no record of human schistosomiasis. Also, some communities registered only altered habitats. All the other urban and rural communities under evaluation presented with all relevant environmental risk factors in addition to notified human cases.

Three sample points did not present relevant malacofauna variables, environmental factors and treated patients (points 26, 27, 43), and were considered to be of low risk for schistosomiasis (Figure 2).

### Environmental Characterization and Additional Risk Factors

In addition to these main variables, environmental, geo-climatic factors, such as temperature, precipitation, altitude and use of vegetation, were analyzed for association with infection.

**Figure 3:**
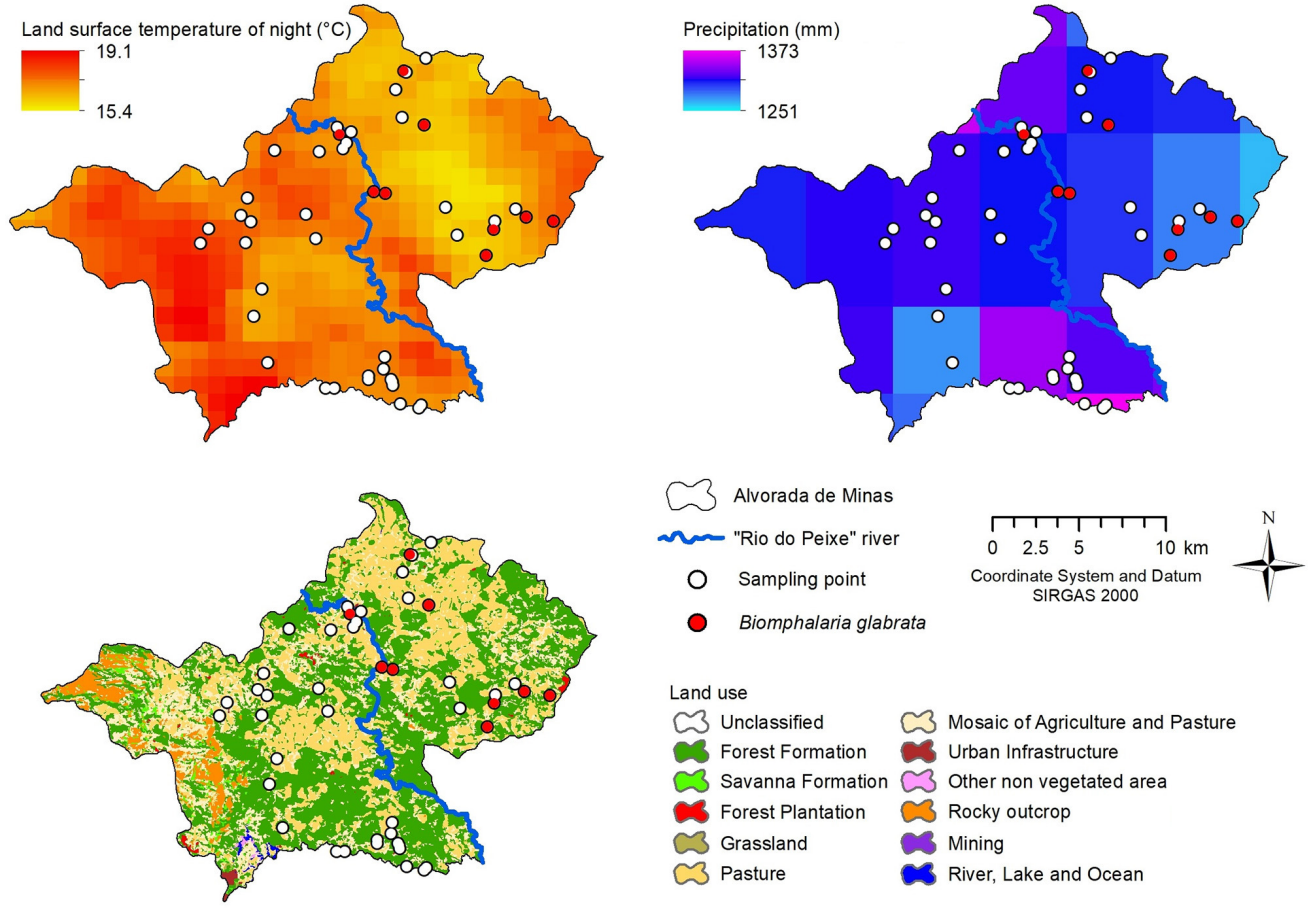
Correlation map of *Biomphalaria glabrata* with precipitation and land surface temperature at night, using 2018–2019 GEE data and land use map, municipality of Alvorada de Minas, Minas Gerais, Brazil.

Pearson's correlation indicated a significant positive correlation with the precipitation variables (indirect) and an inverse correlation with land surface temperature (LST) at night (indirect). The results were: precipitation (cor = 0.36; *p* = 0.01); and LST-night (cor = −0.33; *p* = 0.02) (Figure 3). Other variables did not show significant correlations.

The main river (*Rio do Peixe*) cuts through the central town area from north to south, and is supplied by numerous rivers and streams of small and larger size, whose springs originate from both the western (more rugged relief) and the eastern region (less rugged relief). At lower altitudes, the characteristics of the wavy relief with small plains facilitates the formation of a hydrographic network with favorable backwaters for the establishment of *Biomphalaria*.

The average annual temperature in the municipality varies between 17.8–21.0°C and the annual precipitation varies between 1,358–1,545 mm, with lower temperatures and higher rainfall being observed in the region of higher altitude, in the extreme west, unfavorable for the reproduction of *Biomphalaria*. The lower areas registered higher temperatures and less rainfall, favoring reproduction and establishment of planorbid species.

The land use in Alvorada de Minas is dominated by a fragmented mosaic of “Forest Formation” and “Pasture”, with important continuous and connected forest areas, and large continuous pasture areas with small forest fragments. Other types of land use were also observed, such as “Mosaic of Agriculture and Pasture,” distributed throughout the municipality, and “Rocky Outcrop” and “Savanna Formation,” representative of the quartzitic rock fields of the Serra do Espinhaço in the western region of the municipality. Other types of smaller areas included “Forest Plantation,” “Urban Infrastructure,” and “Mining” and its associated structures. In the western region, there are more natural areas (forest, savanna, and rupestrian fields) in relation to the eastern region of the municipality, which, despite registering important native areas, has extensive pasture areas.

*Biomphalaria glabrata* were predominantly collected at sites that are located close to natural vegetation (forest and savanna−55.56%) and in modified/anthropized areas (pasture and agriculture−44.44%). On the other hand, the opposite was observed at collection points without the presence of planorbid species, where anthropized environments dominated (54.05%).

**Figure 4:**
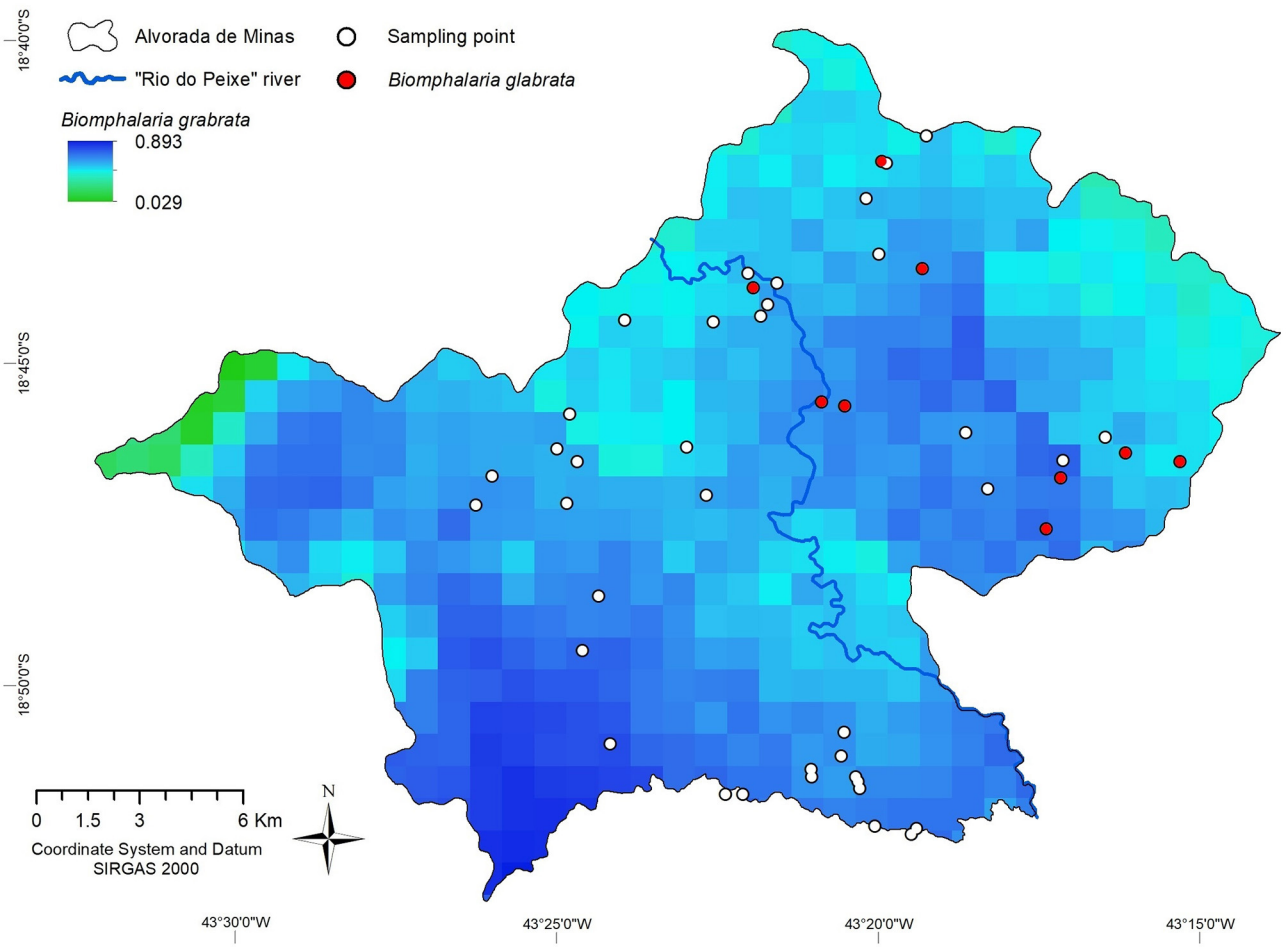
Spatial distribution of *Biomphalaria glabrata* in the municipality of Alvorada de Minas, Minas Gerais, Brazil, obtained by MaxEnt.

The variables which contributed most to the predictive model was the December precipitation (49.2%) and vegetation (21.5%). With an AUC of 0.997, the predictive capacity of the model was high (Figure 4). These variables were in accordance with those found by the correlation (precipitation) and land use/MapBiomas (natural vegetation) models.

### Risk Mapping

**Figure 5:**
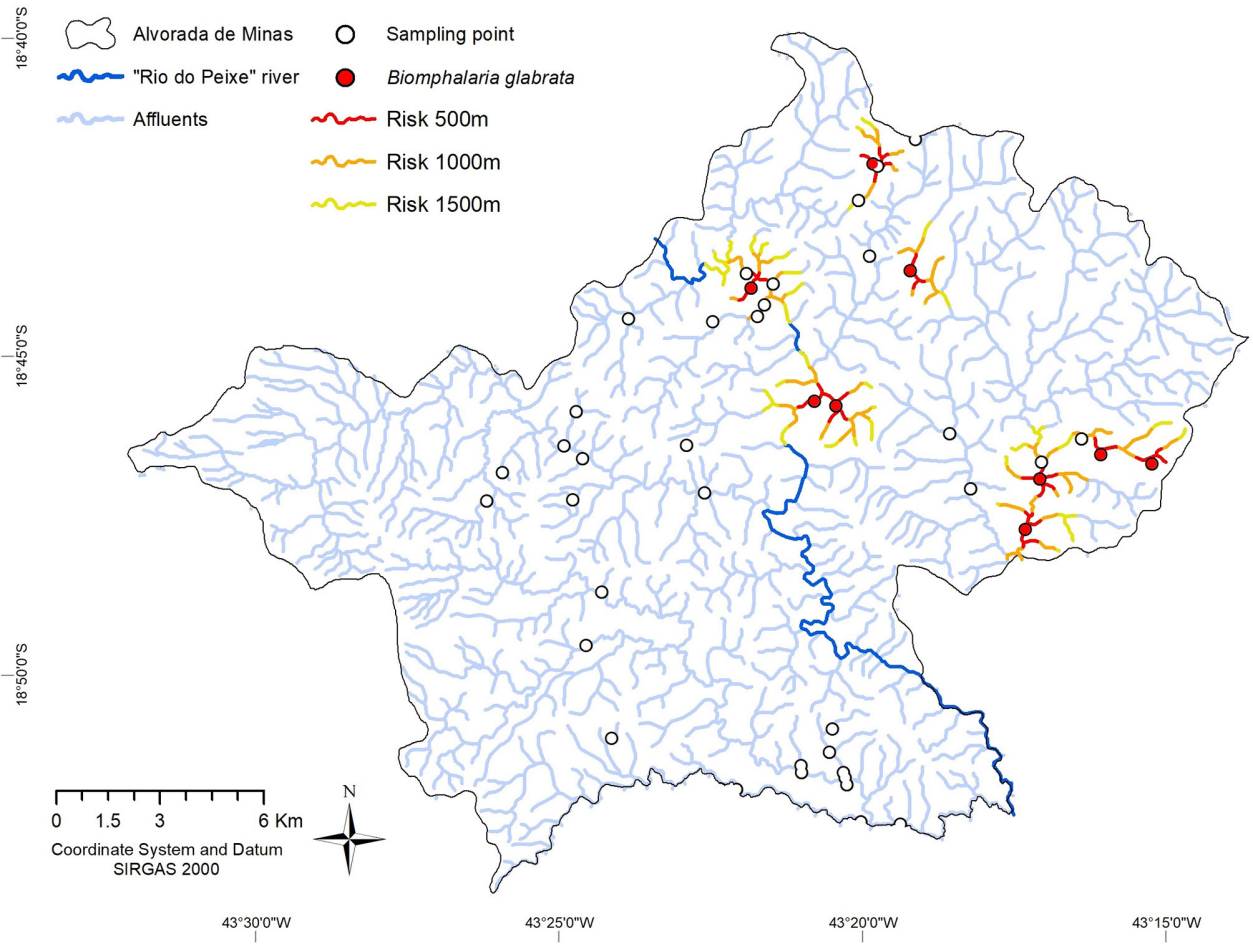
Distribution of *Biomphalaria glabrata* habitats and risk zones, according to Buffer estimates, in the period from 2018–2019, Alvorada de Minas, Minas Gerais State, Brazil. Collection points with *B. glabrata* (red circles) and buffer risk zones for downstream or upstream waterbodies for 500 meters (red), 1,000 meters (orange), and 1,500 meters (yellow) distance.

A buffer map was created to indicate an elevated relative risk in relation to the collection points with *B. glabrata* and within a radius of 500–1,000 m, to indicate areas that demand greater environmental attention in terms of avoidance of human waste disposal. A radius of between 1,000 and 1,500 m was used to indicate the risk of colonization of new environments, considering a potential displacement of *B. glabrata*, passively downstream and actively upstream. Five major stretches of these water resources were identified as of elevated risk for schistosomiasis. The more distant from the point of occurrence, the lower the risk (see Figure 5).

The presence of *B. glabrata* was confirmed at nine sampling points. In the first section located to the east, four points were identified with an approximate route of 7 km in the main channel. The second and third sections were located to the north, with only one point of approximately 3 km in the main channel. The fourth section located in the urban area, also presented a point approximately 300 m from Rio do Peixe and approximately 4 km in the main channel (Rio do Peixe). Finally, the fifth stretch with two points recorded approximately 3.5 km in the main channel (Rio do Peixe). The stretches also reached some of its closest affluents and other points, where no *B. glabrata* were found (see Figure 5).

By the total arbitrary weight technique, we were able to combine additional variables for risk mapping in areas not exclusively associated with the presence of *Biomphalaria*. The total arbitrary weights applied to the sampling points of the limnic environments revealed risk areas other than the risk radius identified in the buffer technique, which was only for *B. glabrata*. Sampling points with the presence of *B. glabrata* registered values of between four and six, suggesting that the presence of these molluscs is strongly associated with at least two other factors analyzed. Some locations registered values of three and two, suggesting potentially favorable environments for *B. glabrata*, e.g., in the district west of the Peixe River. Other locations obtained values of between one and zero, suggesting the absence of environmental conditions for *B. glabrata*, as in the community close to the sampling points 26 and 27 (*Arrudas*), also west of the Peixe River.

**Figure 6:**
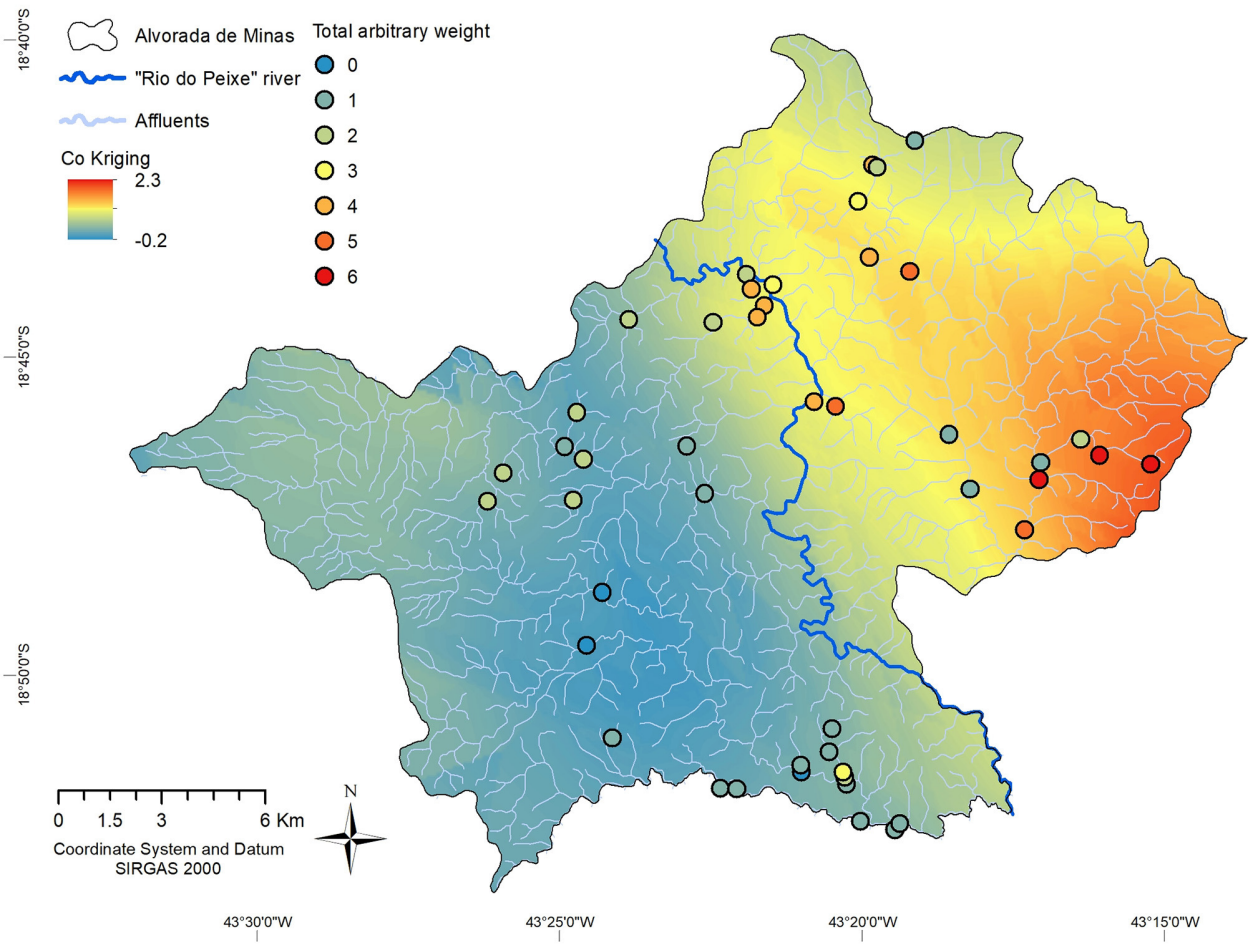
Risk map for transmission of schistosomiasis, estimated by Cokriging with total arbitrary weight of environmental and human epidemiological data, in the Municipality of Alvorada de Minas, Minas Gerais, Brazil. Relative risk is indicated by a graduated color map, with red areas corresponding to high risk and dark blue areas with absent risk of infection.

In order to make better risk predictions, we used cokriging, with the presence of *Biomphalaria* (*B. grabrata* with a value of 2; any other species of *Biomphalaria* value 1) as the main variable and in combination with other support variables, such as community with human cases, altered habitat, and *E. coli* contamination. Cokriging revealed an elevated risk for schistosomiasis (value >) in the main urban area of the municipality and the entire eastern region of Rio do Peixe. Especially in the extreme east, in the Ribeirão community, the risk of infection obtained a value of higher than 2. On the contrary, throughout the western region of the main river, a lower risk for infection (value <1) resulted. The slightly elevated risk attributed in the far west, and the high risk in the far east, both observed beyond the radius of influence of the sampling points, were the result of geostatistical distortions and should be disregarded. The assigned risk estimates are more significant when closer to the sampling points and less powerful in areas with fewer points nearby, e.g., the southern stretch of the Peixe River (Figure 6).

## Discussion and Conclusions

The research carried out analyzed the presence of essential components for the identification of possible risk areas for schistosomiasis (48, 49), such as details on malacofauna, environmental factors related to the disease and the distribution of patients treated by the municipal health department. The integration of this data permitted to create predictive scenarios for the determination of areas with the highest environmental risk, something that was hardly applied in endemic regions in Brazil.

Molecular identification of the collected *Biomphalaria* species was done by PCR-RFLP, a method previously established by our research group in various studies (31, 32). As such, it was shown that the defined restriction profiles are reproducible and proved also to coincide with the morphological identification of the species. For our relatively restricted endemic area with its small but connected water bodies, the objective was to identify the species of the genus *Biomphalaria* and check for the presence of infected snails, without the intention of performing populational studies. In this case, the morphology or/and the PCR-RFLP satisfactorily met our needs.

Among the possible intermediate host species for *S. mansoni* transmission, collected *B. glabrata* species were classified as the most relevant ones, mainly due to their high compatibility with the trematode and their overlap with the distribution of human schistosomiasis in Brazil (50, 51). Furthermore, *B*. glabrata is not only the most susceptible snail species for *S. mansoni* transmission in Brazil, but it is also the species that has the vastest distribution in the State of Minas Gerais (30) and the largest distribution in the municipality of Alvorada de Minas. Consequently, *B. glabrata* was present in nine out of 14 habitats with individuals of the *Biomphalaria* genus, whereas *B. straminea* and *B. tenagophila* were present in only one habitat, that is why, they were included in the arbitrary weight *Biomphalaria* spp. However, under favorable conditions, these snails can transform these environments into places of high risk for transmission.

In the study, more than 80% of the sites with *Biomphalaria* were found to be contaminated with *E. coli* in the limnic environments. In order to investigate the microbiological quality of the water, the microorganisms were used to merely indicate fecal contamination as a kind of surrogate marker, according to the American Public Health Association (APHA) (52). As such, the detection of *E. coli* is indicative of the possible presence of other waterborne pathogens, however, there is no absolute correlation between the number of indicator bacteria and actual presence and the number of enteric pathogens, e.g., schistosome eggs or miracidia (52). According to the WHO (53), *E. coli* is the best microbial indicator of fecal contamination in water samples and is exclusively related to traces of feces, as well as indicating recent fecal contamination in water bodies and can be detected with a low budget. Thus, the presence of bacteria in the aquatic environment may be related to the release of fresh domestic sewage into water bodies or the presence of feces from other homeothermic animals. Nevertheless, in other endemic areas in northeastern Brazil similar results were obtained and indicated the need for improvements in the basic sanitation system of those municipalities (48, 54). Also, from their studies, it was concluded that there was a need to include results on *E. coli* contamination in spatial analyses to contribute to the determination of risk areas. The authors are well aware that there is the possibility of a contribution of other mammals in transmission and maintenance of the biological cycle of *S. mansoni* in endemic areas, mainly by rodent species (55, 56) and by cattle (57–59). However, this was beyond the scope of the present study, which makes it an opportune area for future investigations, since this rural municipality has agriculture as one of its main economic activities.

The protocol for rapid assessment of habitat diversity showed a greater presence of molluscs in impacted stretches, as observed by others during malacological surveys carried out in municipalities of different Brazilian States (60–62). In this respect, others also described the adaptation and strong resistance of different mollusc species, collected in polluted environments (63). Interestingly, human schistosomiasis cases were notified by the local health authorities in the western area. However, no adequate intermediate hosts were found there. Alvorada de Minas is a municipality which is frequented by tourists and backpackers and even for the local population it is relatively easy to get to different locations within the municipality and to adjacent places. As such, we cannot exclude the possibility of dislocation of inhabitants during leisure activities and of water contact in areas where infected molluscs might be present. This justifies the cases of schistosomiasis in the district (Itapanhoacanga) even without the registration of *Biomphalaria* in the western region.

The absence of intermediate hosts in the western region of the investigated municipality may be due to the lower and unfavorable temperatures for *Biomphalaria* in the extreme west. In a similar study, lower temperatures as limiting conditions were also discussed for the distribution of snails and the maturation of cercariae (22). In the same region, the topography presents a considerable elevation (1,300 m above sea level) and the highest parts are mostly rocky, including some mining areas. Thus, the water collections are left without considerable vegetation and not appropriate for the settlement of *Biomphalaria* species. In addition, more elevated springs, more rugged terrain, and higher rainfall result in a higher flow of water, which is a less favorable environment for *Biomphalaria* along the downstream channels, and also reduces the potential for skin contact with released cercariae in such water bodies. As noted by others (64), significant precipitation creates a rapid flow of water unsuitable for cercariae or snail survival. In this same context, the higher flow of water in this region accelerates the purification of water, reducing the chance of fecal contamination by *E. coli*.

In a northeastern state of Brazil, studies used ecological models to determine potential risk areas for the occurrence of *Biomphalaria* species (48, 64). In both studies, precipitation was the variable which contributed most to the predictive models. As for the State of Minas Gerais, rainfall is generally recurrent between October and March (65). Here, the precipitation variable for the month of December was related to the beginning of the rainy period between the months of October 2018 to September 2019. The distribution of snails and, as a consequence, the occurrence of human schistosomiasis cases can be modeled from the environmental and climatic characteristics and, therefore, the *Biomphalaria* distribution maps obtained can be used as risk predictors for the distribution of *S. mansoni*, as previously shown (66).

The Buffer map presented the risk in different circles of risk levels around the collection points with *B. glabrata*, in order to estimate the areas of transmission and the possible expansion to new environments. Other researchers, (67) used the same tool and applied a distance of 100–500 m to determine risk areas in an urbanized, touristic area in Northeastern Brazil. Another study (68), resulted in a significant distance of 400–800 meters from the residences to the water sources defined as risk areas. Thus, demonstrating the importance of such a tool for studies on the epidemiology of schistosomiasis. In general, due to better and more readily available information on the wide distribution of these molluscs and their ability to colonize diverse environments, significantly altered distribution maps on the adequate, intermediate host species for *S. mansoni* transmission were elaborated (69).

In the State of Minas Gerais, some studies showed the potential of using krigging to estimate areas for the occurrence of *Biomphalaria* in designed risk maps (19, 65, 70). In the present study, krigging showed to be a less satisfactory tool, since the relationships between several variables were investigated. In the cokriging system applied here, the cross-correlation between the different variables was more satisfactory to calculate the poorly sampled or unsampled areas, thus providing a tool for the more complete mapping of the risk areas throughout the municipality.

A methodological gap in our study was the use of rough data on human infections from the Municipal Secretary of Health, e.g., merely identification of communities with notified human infections. However, in future investigations on a larger scale, it would be interesting to explore more precisely the spatial effects at the family and/or household level. Another limitation was the lack of an even greater sampling effort of water bodies, which was adjusted to the environments with the greatest potential for occurrence of *Biomphalaria* species and to the vicinity of housings or communities. However, the malacological survey had a good territorial coverage. In our opinion and since schistosomiasis is still considered a major public, economic and environmental health problem, it was important to consider different factors and variables for the determination of risk areas as a kind of integrated approach and based on environmental data and human epidemiology.

For future studies, we would recommend the temporal analysis of risk of infection and the addition of quantitative data on biological materials (examination of human feces; snail data and microbiological exams) collected in the field. Such data collection was beyond the initial scope of the study. However, this could further improve the precision of the model for more accurate estimates of risk areas and its application to identify priority areas for intervention.

Schistosomiasis is a dynamic and resilient disease that predominantly affects impoverished and less developed areas where, in many cases, the parasitic cycle is maintained by a restricted number of affected families or small communities. Alvorada de Minas is a predominantly rural municipality with only one water treatment plant located in the center of the town. The rest of the houses receive water directly from natural water collections without treatment and, in some cases, treated water is distributed by trucks. In this respect, data from the Brazilian Institute of Geography and Statistics from the year 2017, showed that only 33.8% of the households had adequate sanitary sewage, which is considered a risk factor for waterborne diseases (52, 71), especially when combined with the presence of adequate intermediate hosts. As discussed before, the presented analyses helped to identify risk areas. This should give assistance to the municipal decision-making process on the construction of septic tanks and any other sanitation interventions. We believe, that the results of this study may be used to direct measures for improvements in health services and the implementation of control measures for the disease, as well as for social and economical development, since the municipality intends to explore its potential in rural tourism. Spatial analyses must be taken into account for basic sanitation solutions and infrastructure actions, for Alvorada de Minas to develop. This might serve as an example for other, similar communities, which are still endemic for intestinal schistosomiasis.

## Data Availability Statement

The datasets presented in this study can be found in online repositories. The names of the repository/repositories and accession number(s) can be found in the article/Supplementary Material.

## Author Contributions

PC: conception and design of the work, acquisition of data and field work, analysis and interpretation of the work, drafted the work, and critically revised the work. FK: conception and design of the work, acquisition of data, analysis and interpretation of the work, drafted the work, and critically revised the work. AA: data acquisition, analysis and interpretation of the work, and critically revised the work. RG: data acquisition, analysis and interpretation of the work, drafted the work, and critically revised the work. DN-C: data acquisition and fieldwork, analysis and interpretation of the work, and critically revised the work. RC: conception and design of the work, data acquisition, analysis and interpretation of the work, and writing and critical review of the work. SG: conception and design of the work, acquisition of data and field work, analysis and interpretation of the work, drafted the work, and critically revised the work. All authors contributed to the article and approved the submitted version.

## Conflict of Interest

The authors declare that the research was conducted in the absence of any commercial or financial relationships that could be construed as a potential conflict of interest.

## Publisher's Note

All claims expressed in this article are solely those of the authors and do not necessarily represent those of their affiliated organizations, or those of the publisher, the editors and the reviewers. Any product that may be evaluated in this article, or claim that may be made by its manufacturer, is not guaranteed or endorsed by the publisher.

## References

[1] Lutz A. Caramujos de água doce do genero *Planorbis*, observados no Brasil. Mem Inst Oswaldo Cruz. (1918) 10:65–82. 10.1590/S0074-02761918000100004

[2] Noya O Katz N Pontier JP Theron A Noya BA. Schistosomiasis in America. In: Franco-Paredes C Santos-Preciado JI editors. Neglected tropical diseases: Latin America and the Caribbean. Viena: Springer (2015) p. 11–43. 10.1007/978-3-7091-1422-3_2

[3] Colley DG Bustinduy AL Secor WE King CH. Human schistosomiasis. Lancet [Internet]. (2014) 383:2253–64. 10.1016/S0140-6736(13)61949-2 PMC4672382 24698483

[4] Katz N. Inquérito Nacional de Prevalência da Esquistossomose mansoni e Geo-helmintoses (2018). p. 76. Available online at: https://www.arca.fiocruz.br/bitstream/icict/25662/2/InquéritoNacionaldePrevalênciadaEsquistossomosemansonieGeo-helmintoses.pdf

[5] McManus DP Dunne DW Sacko M Utzinger J Vennervald BJ Zhou X-N. Schistosomiasis. Nat Rev Dis Prim [Internet] 9 de dezembro de. (2018) 4:13. 10.1038/s41572-018-0013-8 30093684

[6] Oliveira WJ Magalhães F do C Elias AMS de Castro VN Favero V Lindholz CG. Evaluation of diagnostic methods for the detection of intestinal schistosomiasis in endemic areas with low parasite loads: Saline gradient, Helmintex, Kato-Katz and rapid urine test. PLoS Negl Trop Dis [Internet] 22 de fevereiro de. (2018) 12:e0006232. 10.1371/journal.pntd.0006232 29470516 PMC5823366

[7] Carvalho OS Scholte RGC Guimarães RJPS Freitas CC Drummond SC Amaral RS. The Estrada Real project and endemic diseases: The case of schistosomiasis, geoprocessing and tourism. Mem Inst Oswaldo Cruz. (2010) 105:532–6. 10.1590/S0074-02762010000400031 20721504

[8] Carvalho ODS Mendonça CLF de Marcelino JM da R Passos LKJ Fernandez MA Leal R de S. Distribuição geográfica dos hospedeiros intermediários do *Schistosoma mansoni* nos estados do Paraná, Minas Gerais, Bahia, Pernambuco e Rio Grande do Norte, 2012-2014. Epidemiol e Serv saude Rev do Sist Unico Saude do Bras. (2018) 27(3):e2017343. 10.5123/S1679-49742018000300016 30365698

[9] Rollinson D Knopp S Levitz S Stothard JR Tchuem Tchuenté LA Garba A. Time to set the agenda for schistosomiasis elimination. Acta Trop [Internet]. (2013) 128:423–40. 10.1016/j.actatropica.2012.04.013 22580511

[10] Coura JR Amaral RS. Epidemiological and control aspects of schistosomiasis in Brazilian endemic areas. Mem Inst Oswaldo Cruz. (2004) 99:13–9 10.1590/S0074-02762004000900003 15486629

[11] World Health Assembly 65. Sixty-fifth World Health Assembly, Geneva, 21-26 May 2012: summary records of committees; reports of committees; list of participants. *World Health Organization* (?2012). Available online at: https://apps.who.int/iris/handle/10665/260312

[12] Sokolow SH Wood CL Jones IJ Swartz SJ Lopez M Hsieh MH. Global Assessment of schistosomiasis control over the past century shows targeting the snail intermediate host works best. PLoS Negl Trop Dis. (2016) 10:1–19. 10.1371/journal.pntd.0004794 27441556 PMC4956325

[13] Gray DJ McManus DP Li Y Williams GM Bergquist R Ross AG. Schistosomiasis elimination: lessons from the past guide the future. Lancet Infect Dis [Internet] outubro de. (2010) 10:733–6. 10.1016/S1473-3099(10)70099-2 20705513

[14] Cross ER Sheffield C Perrine R Pazzaglia G. Predicting areas endemic for schistosomiasis using weather variables and a Landsat data base. Mil Med. (1984) 149: 542–544. 10.1093/milmed/149.10.542 6436738

[15] Brown DS. Freshwater Snails of Africa and Their Medical Importance [Internet]. 2nd. ed. CRC Press (1994). p. 321–454. Available online at: https://www.taylorfrancis.com/books/9781482295184

[16] Appleton CC. Review of literature on abiotic factors influencing the distribution and life cycles of bilharziasis intermediate host snails. Malacol Ver. (1978) 11: 1–25.

[17] Bavia ME Malone JB Hale L Dantas A Marroni L Reis R. Use of thermal and vegetation index data from earth observing satellites to evaluate the risk of schistosomiasis in Bahia, Brazil. Acta Trop. (2001) 79:79–85. 10.1016/S0001-706X(01)00105-X 11378144

[18] Freitas C Guimaraes R Dutra L Martins F Gouvea E Santos R. Remote sensing and geographic information systems for the study of schistosomiasis in the state of Minas Gerais, Brazil. In: 2006 IEEE International Symposium on Geoscience and Remote Sensing [Internet]. IEEE (2006). p. 2436–9. 10.1109/IGARSS.2006.631

[19] Guimarães RJPS Freitas CC Dutra L V Felgueiras CA Moura ACM Amaral RS. Spatial distribution of *Biomphalaria* mollusks at São Francisco River Basin, Minas Gerais, Brazil, using geostatistical procedures. Acta Trop [Internet] março de. (2009) 109:181–6. 10.1016/j.actatropica.2008.10.012 19046937

[20] Guimarães RJPS Freitas CC Dutra L V Moura ACM Amaral RS Drummond SC. Schistosomiasis risk estimation in Minas Gerais State, Brazil, using environmental data and GIS techniques. Acta Trop. (2008) 108:234–41. 10.1016/j.actatropica.2008.07.001 18692017

[21] Guimarães RJPS Freitas CC Dutra LV Moura ACM Amaral RS Drummond SC. Analysis and estimative of schistosomiasis prevalence for the state of Minas Gerais, Brazil, using multiple regression with social and environmental spatial data. Mem Inst Oswaldo Cruz. (2006) 101:91–6. 10.1590/S0074-02762006000900014 17308753

[22] Lai YS Biedermann P Ekpo UF Garba A Mathieu E Midzi N. Spatial distribution of schistosomiasis and treatment needs in sub-Saharan Africa: A systematic review and geostatistical analysis. Lancet Infect Dis. (2015) 15:927–40. 10.1016/S1473-3099(15)00066-3 26004859

[23] Ekpo UF Hürlimann E Schur N Oluwole AS Abe EM Mafe MA. Mapping and prediction of schistosomiasis in Nigeria using compiled survey data and Bayesian geospatial modeling. Geospat Health. (2013) 7:355–66. 10.4081/gh.2013.92 23733296

[24] Schur N Hürlimann E Stensgaard AS Chimfwembe K Mushinge G Simoonga C. Spatially explicit *Schistosoma* infection risk in eastern Africa using Bayesian geostatistical modeling. Acta Trop [Internet]. (2013) 128:365–77. 10.1016/j.actatropica.2011.10.006 22019933

[25] Peng WX Tao B Clements A Jiang QL Zhang ZJ Zhou YB. Identifying high-risk areas of schistosomiasis and associated risk factors in the Poyang Lake region, China. Parasitology. (2010) 137:1099–107. 10.1017/S003118200999206X 20128946

[26] Guimarães RJPS Freitas CC Dutra L V Scholte RGC Amaral RS Drummond SC. Evaluation of a linear spectral mixture model and vegetation indices (NDVI and EVI) in a study of schistosomiasis mansoni and *Biomphalaria glabrata* distribution in the state of Minas Gerais, Brazil. Mem Inst Oswaldo Cruz. (2010) 105:512–8. 10.1590/S0074-02762010000400028 20721501

[27] Guimarães RJPS Freitas CC Dutra LV Scholte RGC Flávia TMB Fonseca FR. A geoprocessing approach for studying and controlling schistosomiasis in the state of Minas Gerais, Brazil. Mem Inst Oswaldo Cruz. (2010) 105:524–31. 10.1590/S0074-02762010000400030 20721503

[28] Alvorada de Minas (MG) | Cidades e Estados | IBGE [Internet]. Ibge.gov.br. 2017. Available online at: https://www.ibge.gov.br/cidades-e-estados/mg/alvorada-de-minas.html (accessed 5 Dec, 2020].

[29] Olivier L Schneiderman M A. method for estimating the density of aquatic snail populations. Exp Parasitol [Internet] março de. (1956) 5:109–17. 10.1016/0014-4894(56)90008-X 13317935

[30] Fernandez MA Thiengo SC Amaral RS Técnicas malacológicas. In: Amaral RS Thiengo SC Pieri OS (orgs.). Vigilância e controle de moluscos de importância epidemiológica: diretrizes técnicas. Brasília: Secretaria de Vigilância em Saúde, Ministério da Saúde (2008) p. 43–70.

[31] Vidigal THDA Caldeira RL Simpson AJG Carvalho OS. Further studies on the molecular systematics of *Biomphalaria* snails from Brazil. Mem Inst Oswaldo Cruz. (2000) 95:57–66. 10.1590/S0074-02762000000100009 10656706

[32] Caldeira RL Teodoro TM Jannotti-Passos LK Lira-Moreira PM Goveia CDO Carvalho ODS. Characterization of South American snails of the genus *Biomphalaria* (Basommatophora: Planorbidae) and *Schistosoma mansoni* (Platyhelminthes: Trematoda) in molluscs by PCR-RFLP. Biomed Res Int. (2016) 2016 10.1155/2016/1045391 PMC5131227 27981045

[33] Deslandes N. Técnica de dissecção e exame de planorbídeos. Rev Saúde Pbl. (1951) 4:371–82.

[34] Paraense WL. Estado atual da sistemática dos planorbídeos brasileiros. Arq Mus Nac Rio de Janeiro. (1975) 55:105–28.

[35] Kuntz ER. Effect of light and temperature on shedding of *Schistosoma mansoni* cercariae. Naval Medical Research Institute. (1946) 7:16.

[36] Jannotti-Passos LK Caldeira RL Carvalho OS. Técnicas utilizadas no estudo dos moluscos do gênero *Biomphalaria* e na manutenção do ciclo de *Schistosoma mansoni*. In: Carvalho OS Coelho PMZ Lenzi HL. Schistosoma mansoni e esquistossomose: uma visão multidisciplinar. Rio de Janeiro: Fiocruz (2008) p. 531–44.

[37] Center for Food Safety and Applied Nutrition. BAM Chapter 4 [Internet]. U.S. Food and Drug Administration. 2020. Available online at: https://www.fda.gov/food/laboratory-methods-food/bam-chapter-4-enumeration-escherichia-coli-and-coliform-bacteria (accessed Dec7, 2020)

[38] Kämpfer P Nienhüser A Packroff G Wernicke F Mehling A Nixdorf K. Molecular identification of coliform bacteria isolated from drinking water reservoirs with traditional methods and the Colilert-18 system. Int J Hyg Environ Health [Internet] julho de. (2008) 211:374–84. 10.1016/j.ijheh.2007.07.021 17870668

[39] Fricker EJ Illingworth KS Fricker CR. Use of two formulations of Colilert and QuantiTray^TM^ for assessment of the bacteriological quality of water. Water Res [Internet] outubro de. (1997) 31:2495–9. 10.1016/S0043-1354(96)00342-9

[40] Biological Criteria National Program Guidance For Surface Waters [Internet]. Available online at: https://www.epa.gov/sites/production/files/2018-10/documents/national-program-guidance-surface_waters.pdf (accessed Dec7, 2020)

[41] Callisto M Ferreira WR Moreno P Goulart M Petrucio M. Aplicação de um protocolo de avaliação rápida da diversidade de habitats em atividades de ensino e pesquisa (MG-RJ). Acta Limnol Bras. (2002) 14:91–8.

[42] Brito MTS Diniz LP Silva-Cavalcanti JS Melo Júnio M. Protocolos de avaliação ambiental rápida de açudes do Semiárido: Adaptações regionais e um estudo de caso (2011) In: Messias AS Feitosa MCA. (Org.) A influência das mudanças climáticas sobre os recursos hídricos. 6° Edição. Recife: UNICAP. (2011) p. 130–137.

[43] Gorelick N. Hancher, M, Dixon M, Ilyushenko S, Tchau D, Moore R. Google Earth Engine: Planetary-scale geospatial analysis for everyone. Remote Sensing of Environment, v 202, n. (2017) 1:18–27. 10.1016/j.rse.2017.06.031

[44] Souza CM Z Shimbo J Rosa MR Parente LL A Alencar A Rudorff BFT. Reconstructing three decades of land use and land cover changes in Brazilian biomes with landsat archive and earth engine. Remote Sens [Internet] 25 de agosto de. (2020) 12:2735. 10.3390/rs12172735

[45] Santos SM Souza WV (Orgs.). Introdução à Estatística Espacial para a Saúde Pública. Brasília: Ministério da Saúde (2007) p. 120

[46] Câmara G Carvalho MS Cruz OG Correa V 2004. Análise espacial de áreas. In: Embrapa Cerrados. Análise espacial de dados geográficos. Planaltina, DF: Embrapa; (2004). p.157–209.

[47] Teodoro TM Janotti-Passos LK Carvalho Odos S Caldeira RL. Occurrence of *Biomphalaria cousini* (Mollusca: Gastropoda) in Brazil and its susceptibility to *Schistosoma mansoni* (Platyhelminths: Trematoda). Mol Phylogenet Evol. (2010) 57:144–51. 10.1016/j.ympev.2010.05.019 20580934

[48] Gomes ECS Mesquitta MCS Wanderley L de Melo F. de Paula Souza e Guimarães R, Barbosa C. Spatial risk analysis on occurrences and dispersal of Biomphalaria straminea in and endemic area for schistosomiasis. J Vector Borne Dis [Internet]. (2018) 55:208. 10.4103/0972-9062.249142 30618447

[49] Barbosa CS Favre TC Amaral RS Pieri OS. Epidemiologia e Controle da Esquistossomose Mansônica. In: Carvalho OS Coelho PMZ Lenzi HL. Schistosoma mansoni e esquistossomose: uma visão multidisciplinar. Rio de Janeiro: Editora Fiocruz. (2008) p. 965–1008.

[50] Souza CP de Jannotti-Passos LK Freitas JR de. Degree of host-parasite compatibility between *Schistosoma mansoni* and their intermediate molluscan hosts in Brazil. Mem Inst Oswaldo Cruz [Internet] fevereiro de. (1995) 90:5–10. 10.1590/S0074-02761995000100003 8524084

[51] Brasil. Ministério da Saúde. Secretaria de Vigilância em Saúde. Departamento de Vigilância Epidemiológica. Vigilância da Esquistossomose Mansoni: diretrizes técnicas / Ministério da Saúde, Secretaria de Vigilância em Saúde, Departamento de Vigilância das Doenças Transmissíveis. 4th edn Brasília: Ministério da Saúde. (2014) p. 144

[52] American Public Health Association (APHA). Standard Methods for Examination of Water and Wastewater. 22. ed. Washington, D.C.: APHA. (2012).

[53] World Health Organization (WHO). Guidelines for Drinking-water Quality. 4. edn. Geneva: WHO Library. (2011).

[54] Bezerra FS de M Pinheiro MCC Silva Filho JD da Castro IMN de Caldeira RL Sousa MS. Identification of *Biomphalaria sp*. and other freshwater snails in the large-scale water transposition project in the Northeast of Brazil. Rev Inst Med Trop Säo Paulo [Internet]. 20 de agosto de. (2018) 60. 10.1590/s1678-9946201860041 30133601 PMC6103326

[55] Martins AV Martins G Brito RS. Wild reservoirs of the Schistosoma mansoni in the State of Minas Gerais. Rev Brasil Malariol Doencas Trop. (1955) 7: 259–25. 13310897

[56] Miranda GS Miranda BS Rodrigues JGM Lira MGS Nogueira RA Melo DV. Research Note. The wild water-rats and their relevance in the context of schistosomiasis mansoni in Brazil: what we know and recommendations for further research. Helminthologia, v. (2017) 54:1–5. 10.1515/helm-2017-0013

[57] Barbosa FS Barbosa I Arruda F. *Schistosoma mansoni*: natural infection of cattle in Brazil. Science. (1962) 138:831 10.1126/science.138.3542.831 17821007

[58] Coelho PMZ Nogueira RHG Lima WS Cunha MC. *Schistosoma mansoni*:experimental bovine schistosomiasis. Rev Inst Med Trop. (1982) 24:374–7. 7182913

[59] Modena CM Lima WS Coelho PMZ Barbosa FS. Aspectos epidemiológicos da esquistossomose mansoni em bovinos. Arch Bras Med Vet Zootec. (1991) 43:481–8.

[60] Souza MAA Barbosa VS Albuquerque JO Bocanegra S Souza-Santos R Paredes H. Aspectos ecológicos e levantamento malacológico para identificação de áreas de risco para transmissão da esquistossomose mansoni no litoral norte de Pernambuco, Brasil. Iheringia Série Zool [Internet] 30 de março de. (2010) 100:19–24. 10.1590/S0073-47212010000100003

[61] Souza MAA de Barbosa VS Wanderlei TNG Barbosa CS. Criadouros de *Biomphalaria*, temporários e permanentes, em Jaboatão dos Guararapes, PE. Rev Soc Bras Med Trop [Internet] junho de. (2008) 41:252–6. 10.1590/S0037-86822008000300006 18719804

[62] Souza MAA de Souza LA de Machado-Coelho GLL Melo AL de. Levantamento malacológico e mapeamento das áreas de risco para transmissão da esquistossomose mansoni no Município de Mariana, Minas Gerais, Brasil. Rev Ciências Médicas e Biológicas [Internet]. 19 de janeiro de. (2006) 5. 10.9771/cmbio.v5i2.4120

[63] Moreno P Callisto M. Benthic Macroinvertebrates in the Watershed of an Urban Reservoir in Southeastern Brazil. Hydrobiologia [Internet]. (2006) 560:311–21. 10.1007/s10750-005-0869-y

[64] McCreesh N Booth M. Challenges in predicting the effects of climate change on *Schistosoma mansoni* and Schistosoma haematobium transmission potential. Trends Parasitol [Internet] novembro de. (2013) 29:548–55. 10.1016/j.pt.2013.08.007 24064438

[65] Catalunha MJ Sediyama GC Leal BG Soares CPB Ribeiro A. Aplicação de cinco funções de densidade de probabilidade a séries de precipitação pluvial no Estado de Minas Gerais. Revista Brasileira de Agromeleorologia: Santa Maria. (2002) p. 153–162.

[66] Barbosa VS E Guimarães RJ de PS Loyo RM Barbosa CS. Modeling of the distribution of *Biomphalaria glabrata* and *Biomphalaria straminea* in the metropolitan region of Recife, Pernambuco, Brazil. Geospat Health [Internet]. 25 de novembro de. (2016) 11. 10.4081/gh.2016.490 27903064

[67] Barbosa CS Souza ATOF Leal Neto OB Gomes ECS Araujo KCGM Guimarães RJPS. e. Turismo de risco para esquistossomose mansônica em Porto de Galinhas, Estado de Pernambuco, Brasil. Rev Pan-Amazônica Saúde [Internet] setembro de. (2015) 6:51–8. 10.5123/S2176-62232015000300007

[68] Clennon JA King CH Muchiri EM Kariuki HC Ouma JH Mungai P. Spatial patterns of urinary schistosomiasis infection in a highly endemic area of coastal Kenya. Am J Trop Med Hyg. (2004) 70:443–48. 10.4269/ajtmh.2004.70.443 15100462

[69] Brasil MS. Ministério da Saúde. Secretaria de Vigilância em Saúde. Departamento de Vigilância Epidemiológica. Vigilância e Controle de Moluscos de Importância Epidemiológica - Diretrizes técnicas: Programa de Vigilância e Controle da Esquistossomose (PCE). Editora do Ministério da Saúde, organizador. Série A. Brasília – DF: Normas e Manuais Técnicos. (2008) p. 178.

[70] Guimarães RJPS Freitas CC Dutra L V Felgueiras CA Drummond SC Tibiriçá SHC. Use of Indicator Kriging to Investigate Schistosomiasis in Minas Gerais State, Brazil. J Trop Med [Internet]. (2012) 2012:1–10. 10.1155/2012/837428 22291716 PMC3265113

[71] Ahmed W Sidhu JPS Smith K Beale D Gyawali P Toze S. Distributions of fecal markers in wastewater from different climatic zones for human fecal pollution tracking in Australian surface waters. Appl Environ Microbiol. (2015) 4:1316–23. 10.1128/AEM.03765-15 26682850 PMC4751839

