# Supplementary material for: Identification of Risk Areas for Intestinal Schistosomiasis, Based on Malacological and Environmental Data and on Reported Human Cases
Source: Front Med (Lausanne). 2021 Aug 6;8:642348. doi: 10.3389/fmed.2021.642348 (PMC8377395; doi:10.3389/fmed.2021.642348)
Supplement: Supplementary file 1 [file Data_Sheet_1.docx]

APPENDIX:

Appendix 1 - Protocol for rapid assessment of habitat diversity in stretches of river basins^+)^.

| Parameter | SCORING SYSTEM | | |
| --- | --- | --- | --- |
|  | Score 4 | Score 2 | Score 0 |
| 1. Type of occupation of the margins of the water body (main activity) | Natural vegetation | Pasture, agriculture, monoculture, reforestation | Residential, commercial and/or industrial |
| 2. Erosion near and/or on the banks of the river and silting up in its bed | Absent | Moderate | Sharp |
| 3. Human changes | Absent | Changes of domestic origin (sewage, garbage) | Changes of urban industrial origin |
| 4. Vegetation covering of the river bed | Partial | Total | Absent |
| 5. Water odor | None | Seaweed and/or sewage | Oil/ Industrial |
| 6. Water greasiness | Absent | Moderate | Abundant |
| 7. Water color | Transparent | Blurred / strong tea color | Opaque or colored |
| 8. Presence of aquatic plants | Abundant | Moderate | Absent |
| 9. Type of fund | Stones / gravel | Mud / sand | Cement / piped |
| 10. Habitat diversity | Stable for maintaining aquatic communities | Insufficient availability | Absence of habitats |

+) Adapted and modified from Callisto *et al*. (2002).

Appendix 2: Epidemiological, biological and environmental data obtained in the field in the municipality of Alvorada de Minas, Minas Gerais, Brazil.

| Locations | Points | *Biomphalaria* spp. | *B. glabrata* | *B. straminea* | *B. tenagophila* | *B. cousini* | *B. kuhniana* | Total coliforms | *E. coli* | Habitat Assessment Score | Patients diagnosed and treated |
| --- | --- | --- | --- | --- | --- | --- | --- | --- | --- | --- | --- |
| Ribeirão | 1 | X | X |  |  |  | X | X | X | 20 | X |
|  | 2 | X | X |  |  |  | X | X | X | 16 | X |
|  | 3 |  |  |  |  |  |  |  |  | 8 | X |
|  | 4 |  |  |  |  |  |  |  |  | 32 | X |
|  | 5 | X | X | X |  |  | X | X | X | 20 | X |
|  | 6 | X | X |  |  |  |  | X | X | 20 | X |
| Hydrographic basin Bibiu | 7 |  |  |  |  |  |  |  |  | 10 |  |
|  | 8 |  |  |  |  |  |  |  |  | 8 |  |
| Fazenda da Ponte | 9 | X | X |  |  |  |  | X | X | 12 | X |
|  | 10 | X | X |  |  |  |  | X | X | 28 | X |
| Centro | 11 | X |  |  |  |  | X | X | X | 16 | X |
|  | 12 | X |  |  |  |  |  | X | X | 18 | X |
|  | 13 |  |  |  |  |  |  | X | X | 18 | X |
|  | 14 |  |  |  |  |  |  | X | X | 28 | X |
|  | 15 | X | X |  |  |  |  | X | 0 | 18 | X |
|  | 16 |  |  |  |  |  |  |  |  | 14 | X |
| Bom Jesus | 17 |  |  |  |  |  |  |  |  | 20 | X |
| Barbeiro | 18 |  |  |  |  |  |  | X | X | 26 |  |
| Hydrographic basin escadinha | 19 |  |  |  |  |  |  |  |  | 28 | X |
|  | 20 |  |  |  |  |  |  |  |  | 18 | X |
|  | 21 |  |  |  |  |  |  |  |  | 18 | X |
|  | 22 |  |  |  |  |  |  |  |  | 10 | X |
|  | 23 |  |  |  |  |  |  | X | X | 28 | X |
|  | 24 |  |  |  |  |  |  |  |  | 4 | X |
| Alto dos Monteiros | 25 |  |  |  |  |  |  | N.D. | N.D. | 18 |  |
| Hydrographic basin São José | 26 |  |  |  |  |  |  |  |  | 30 |  |
|  | 27 |  |  |  |  |  |  |  |  | 36 |  |
| Água Quente | 28 |  |  |  |  |  |  |  |  | 20 |  |
|  | 29 |  |  |  |  |  |  |  |  | 20 |  |
| Jassém | 30 |  |  |  |  |  |  |  |  | 18 |  |
|  | 31 |  |  |  |  |  |  |  |  | 12 |  |
|  | 32 | X |  |  |  |  |  | X | 0 | 18 |  |
|  | 33 | X |  |  | X |  |  | X | X | 16 |  |
|  | 34 |  |  |  |  |  |  |  |  | 8 |  |
|  | 35 |  |  |  |  |  |  |  |  | 8 |  |
| Hydrographic basin Pinheiros | 36 |  |  |  |  |  |  |  |  | 16 |  |
| Lambari | 37 | X | X |  |  |  |  | N.D. | N.D. | 16 | X |
|  | 38 |  |  |  |  |  |  |  |  | 10 | X |
|  | 39 |  |  |  |  |  |  | X | X | 18 | X |
|  | 40 | X |  |  |  | X |  | X | X | 14 | X |
|  | 41 | X | X |  |  |  | X | X | 0 | 10 | X |
| Hydrographic basin Pau Lavrado | 42 |  |  |  |  |  |  |  |  | 28 |  |
| Jassém | 43 |  |  |  |  |  |  |  |  | 12 |  |
|  | 44 |  |  |  |  |  |  |  |  | 20 |  |
|  | 45 |  |  |  |  |  |  |  |  | 20 |  |
|  | 46 |  |  |  |  |  |  |  |  | 20 |  |

X: Present; 0: *E. coli* absent; N.D.: No Data.
